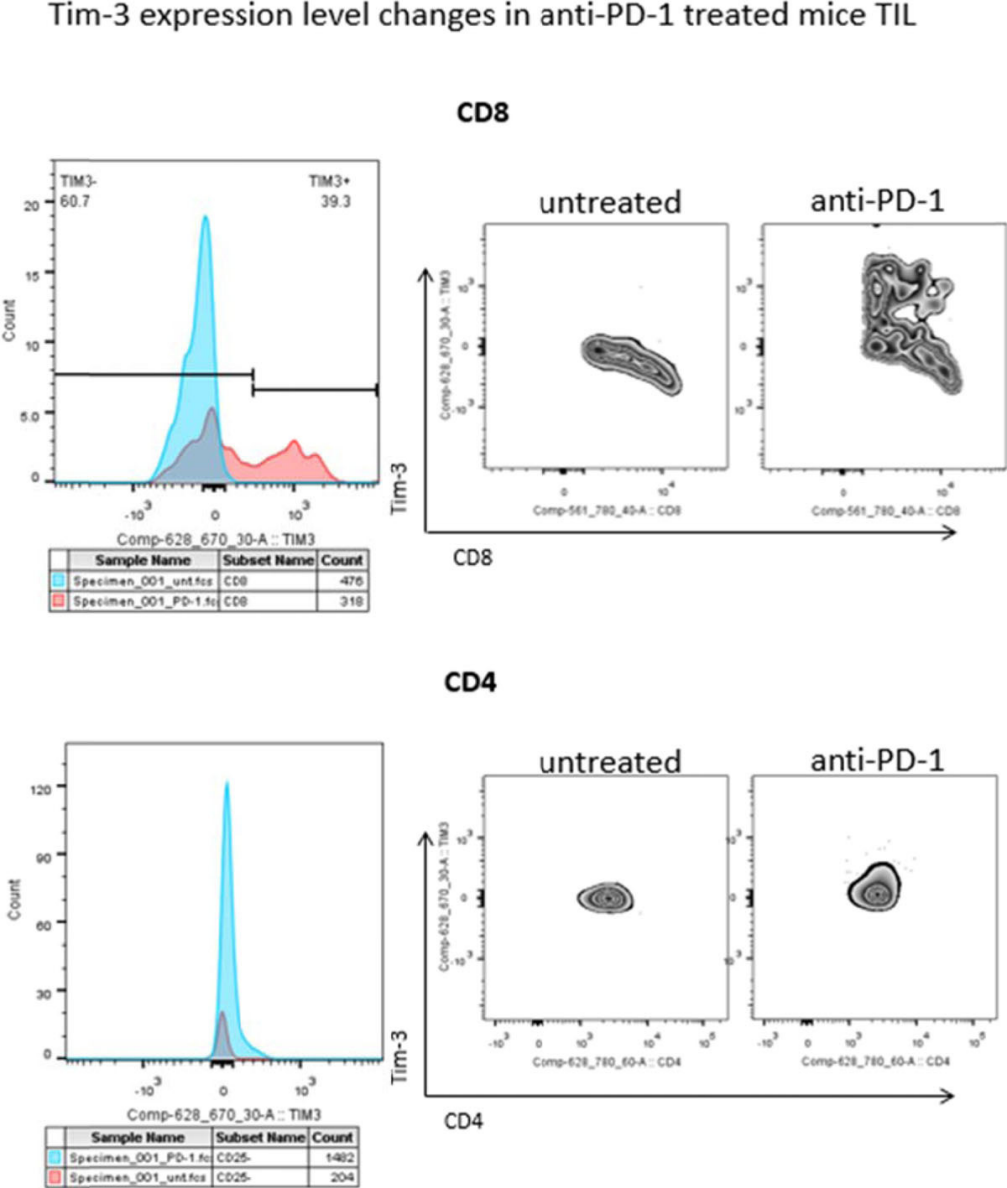# PD-1 blockade upregulate TIM-3 expression as a compensatory regulation of immune check point receptors in HNSCC TIL

**DOI:** 10.1186/2051-1426-3-S2-P196

**Published:** 2015-11-04

**Authors:** Gulidanna Shayan, Robert L Ferris

**Affiliations:** 1 University of Pittsburgh Hillman Cancer Center, Pittsburgh, PA, USA; 2 University of Pittsburgh Cancer Institute, Pittsburgh, PA, USA

## 

Programmed Death 1 (PD-1) and T cell Ig and mucin domain-3 protein (Tim-3) are immune check point receptors that are upregulated on tumor infiltrating lymphocytes (TIL) in tumor-bearing mice and humans. As anti-PD-1 single agent response rates are still relatively low (20%) in HNC patients, it is important to learn how different inhibitory check point receptors work together to maintain the suppressive status of immune system. We observed that PD-1 and Tim-3 co-expression is associated with an exhausted phenotype of HNSCC TIL of patients, demonstrating the highest PD-1 levels in Tim-3 double positive TIL. We also observed that PD-1+/Tim-3+ TIL manifest dampened in Akt/p-S6 activation upon TCR stimulation, leading us to infer the potential for signaling cross-talk between PD-1 and Tim-3 downstream signaling pathways. Indeed, in freshly isolated HNSCC TIL, PD-1-/TIM-3+ T cells showed higher baseline expression of p-SHP-2 (p < 0.01) which is triggered upon PD-1 ligation. In addition, PD-1 blockade using nivolumab of human HNSCC TIL led to upregulation of Tim-3 expression, suggesting a circuit of compensatory, cross-talk signaling and permitting escape from anti-PD-1 blockade during TCR stimulation. In a murine HNC tumor model, anti-PD-1 treatment modestly suppressed tumor growth, but in TIL from persistent tumors in these mice, Tim-3 expression was dramatically upregulated after PD-1 blockade. Taken together, in response to PD-1 blockade, the most exhausted TIL appear to upregulate Tim-3 as a compensatory mechanism, supporting dual targeting, which may provide new therapeutic strategy for cancer immunotherapy.

**Figure 1:**
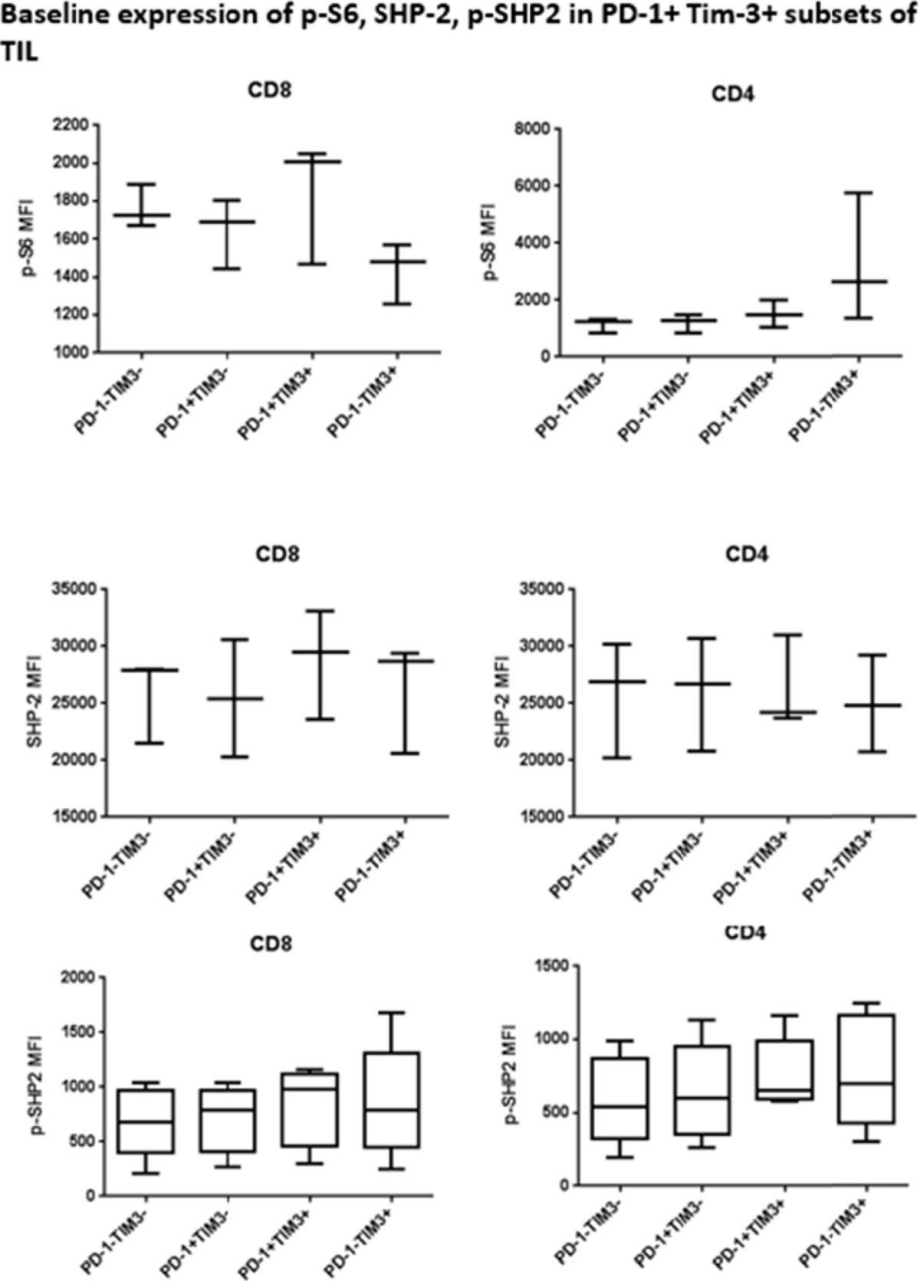


**Figure 2:**
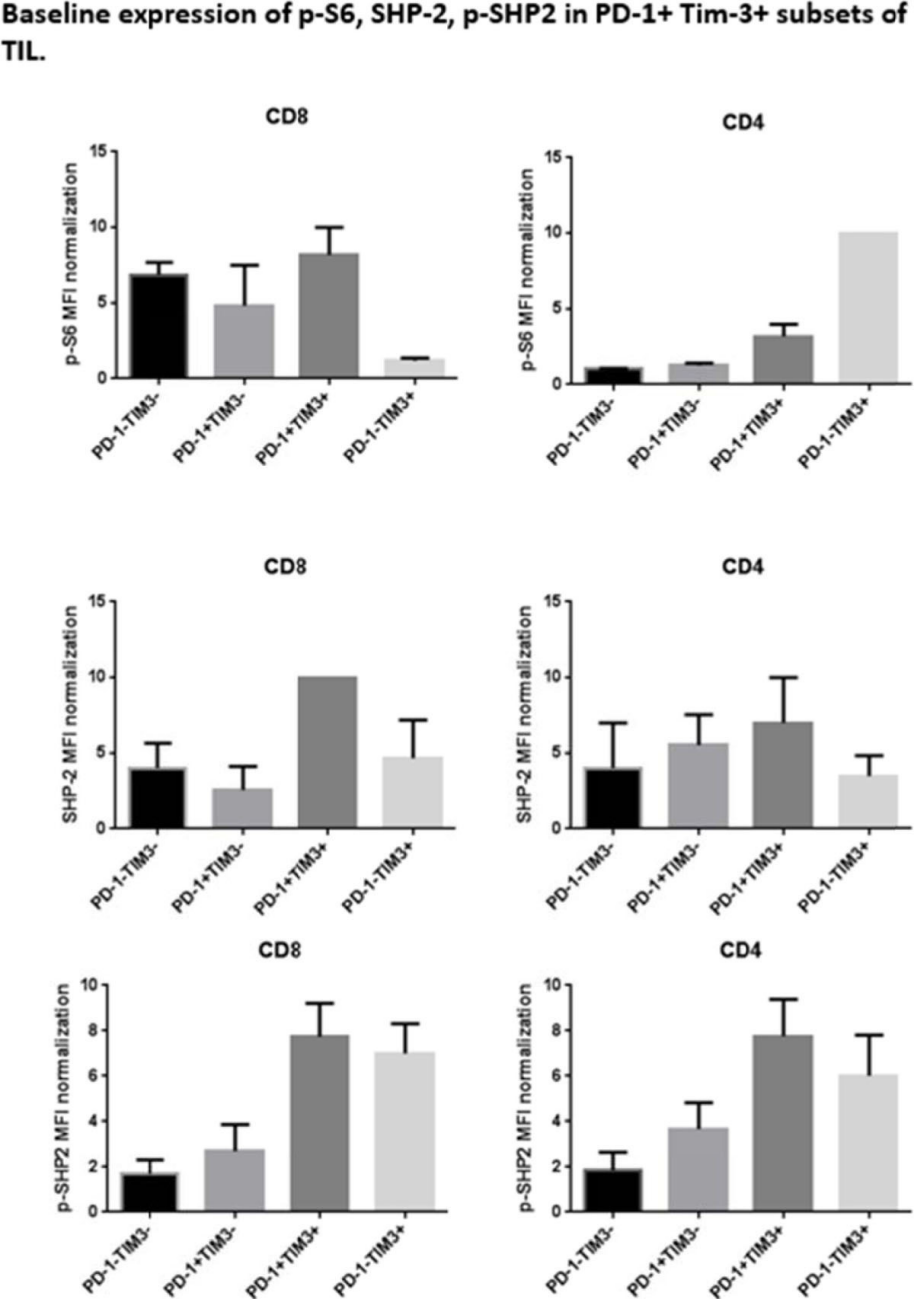


**Figure 3:**
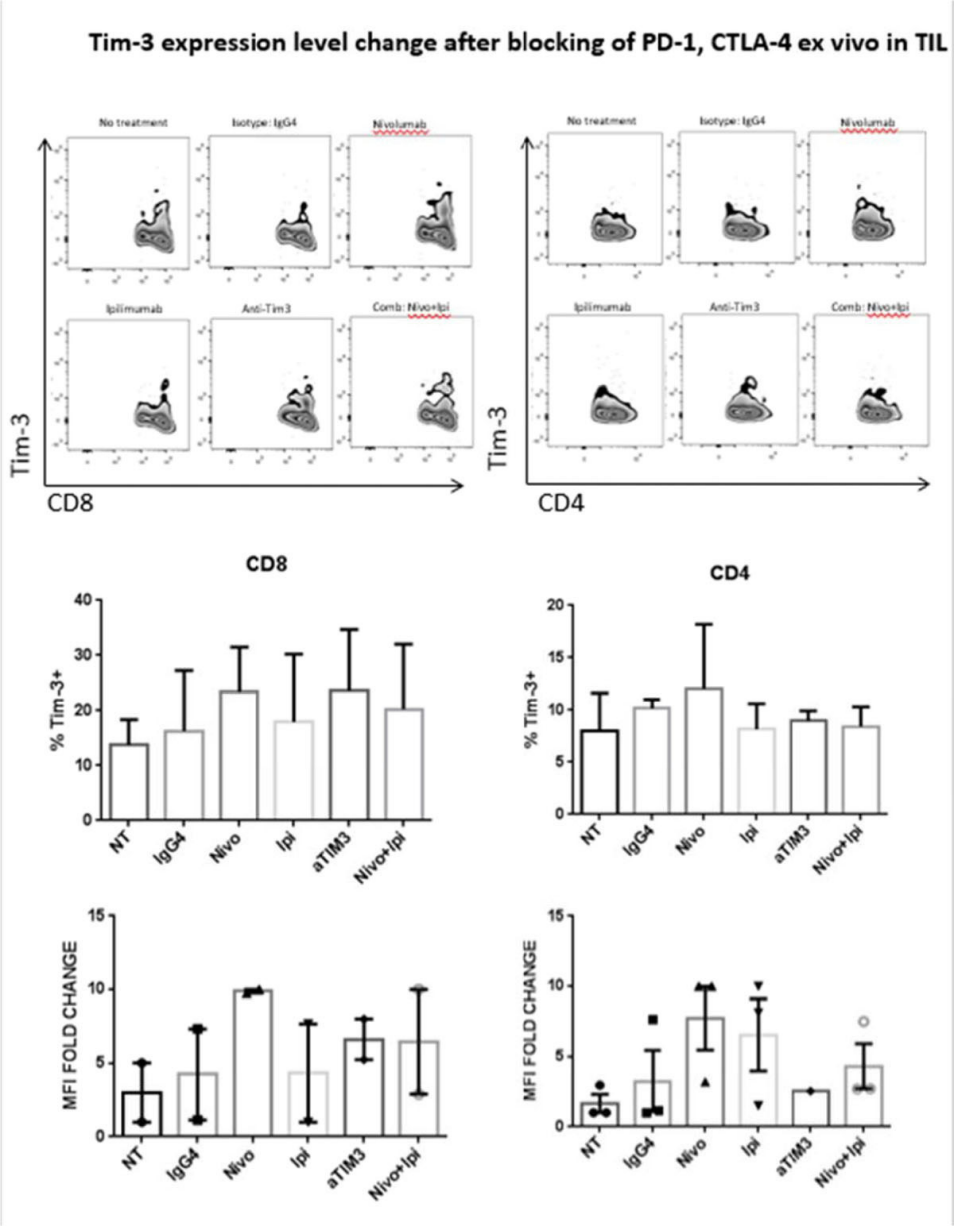


**Figure 4:**